# Comparison of quality-of-care measures in U.S. patients with end-stage renal disease secondary to lupus nephritis vs. other causes

**DOI:** 10.1186/s12882-015-0037-1

**Published:** 2015-03-29

**Authors:** Laura C Plantinga, Rachel E Patzer, Cristina Drenkard, Stephen O Pastan, Jason Cobb, William McClellan, Sung Sam Lim

**Affiliations:** Department of Medicine, Emory University, Atlanta, Georgia USA; Department of Surgery, Emory University, Atlanta, Georgia USA; Emory Transplant Center, Emory Healthcare, Atlanta, Georgia USA; Department of Epidemiology, Emory University, Atlanta, Georgia USA

## Abstract

**Background:**

Patients with end-stage renal disease (ESRD) due to lupus nephritis (LN-ESRD) may be followed by multiple providers (nephrologists and rheumatologists) and have greater opportunities to receive recommended ESRD-related care. We aimed to examine whether LN-ESRD patients have better quality of ESRD care compared to other ESRD patients.

**Methods:**

Among incident patients (7/05–9/11) with ESRD due to LN (*n* = 6,594) vs. other causes (*n* = 617,758), identified using a national surveillance cohort (United States Renal Data System), we determined the association between attributed cause of ESRD and quality-of-care measures (pre-ESRD nephrology care, placement on the deceased donor kidney transplant waitlist, and placement of permanent vascular access). Multivariable logistic and Cox proportional hazards models were used to estimate adjusted odds ratios (ORs) and hazard ratios (HRs).

**Results:**

LN-ESRD patients were more likely than other ESRD patients to receive pre-ESRD care (71% vs. 66%; OR = 1.68, 95% CI 1.57-1.78) and be placed on the transplant waitlist in the first year (206 vs. 86 per 1000 patient-years; HR = 1.42, 95% CI 1.34–1.52). However, only 24% had a permanent vascular access (fistula or graft) in place at dialysis start (vs. 36%; OR = 0.63, 95% CI 0.59–0.67).

**Conclusions:**

LN-ESRD patients are more likely to receive pre-ESRD care and have better access to transplant, but are less likely to have a permanent vascular access for dialysis, than other ESRD patients. Further studies are warranted to examine barriers to permanent vascular access placement, as well as morbidity and mortality associated with temporary access, in patients with LN-ESRD.

## Background

Among end-stage renal disease (ESRD) patients, receipt of pre-ESRD care [1-9], access to kidney transplantation [10-15], and permanent vascular accesses for dialysis, which include arteriovenous fistulae (AVFs) and grafts [16-23], are all associated with better patient outcomes and lower healthcare costs. Benchmarks for ESRD healthcare quality are provided in Healthy People 2020 (www.healthypeople.gov) [24]. Further, Centers for Medicare & Medicaid Services (CMS) is incentivized to promote quality of care due to universal coverage of ESRD care for all eligible U.S. patients. Accordingly, CMS mandates ESRD pay-for-performance [25] and quality improvement projects addressing quality of ESRD care that are regionally implemented through its 18 ESRD Networks. Since 2005, CMS has also collected information on quality-of-ESRD-care measures on all incident ESRD patients via the CMS Medical Evidence Report (CMS Form 2728), which is completed for all patients at the start of ESRD treatment.

Recently, we reported on the sociodemographic and geographic predictors of quality of ESRD care in the population with ESRD attributed to lupus nephritis (LN-ESRD) [26], and others have reported on placement on the deceased donor kidney transplant waitlist among these patients [27,28]. However, the translation of these measures among LN-ESRD patients has not been compared to that among other ESRD patients. Translation of quality-of-care measures should be as good as, or better, in patient populations treated by multiple specialty providers, such as those with LN-ESRD, relative to the overall population. However, a similar U.S. population of ESRD patients in terms of age and race as well as receipt of multi-provider treatment—those with ESRD secondary to sickle cell disease—was shown to have poorer quality of care than patients with ESRD due to other causes [29]. A comparison among patients with ESRD due to LN vs. other causes is important because nephrologists could partner with rheumatologists, who currently have few guidelines to address the preparation for ESRD among their systemic lupus erythematosus (SLE) patients [30], and other providers to address identified gaps in the quality of ESRD care among these patients. Thus, we sought to compare the translation of ESRD quality-of-care measures among U.S. patients with LN-ESRD vs. ESRD due to other causes.

## Methods

### Study population and data sources

**Figure 1:**
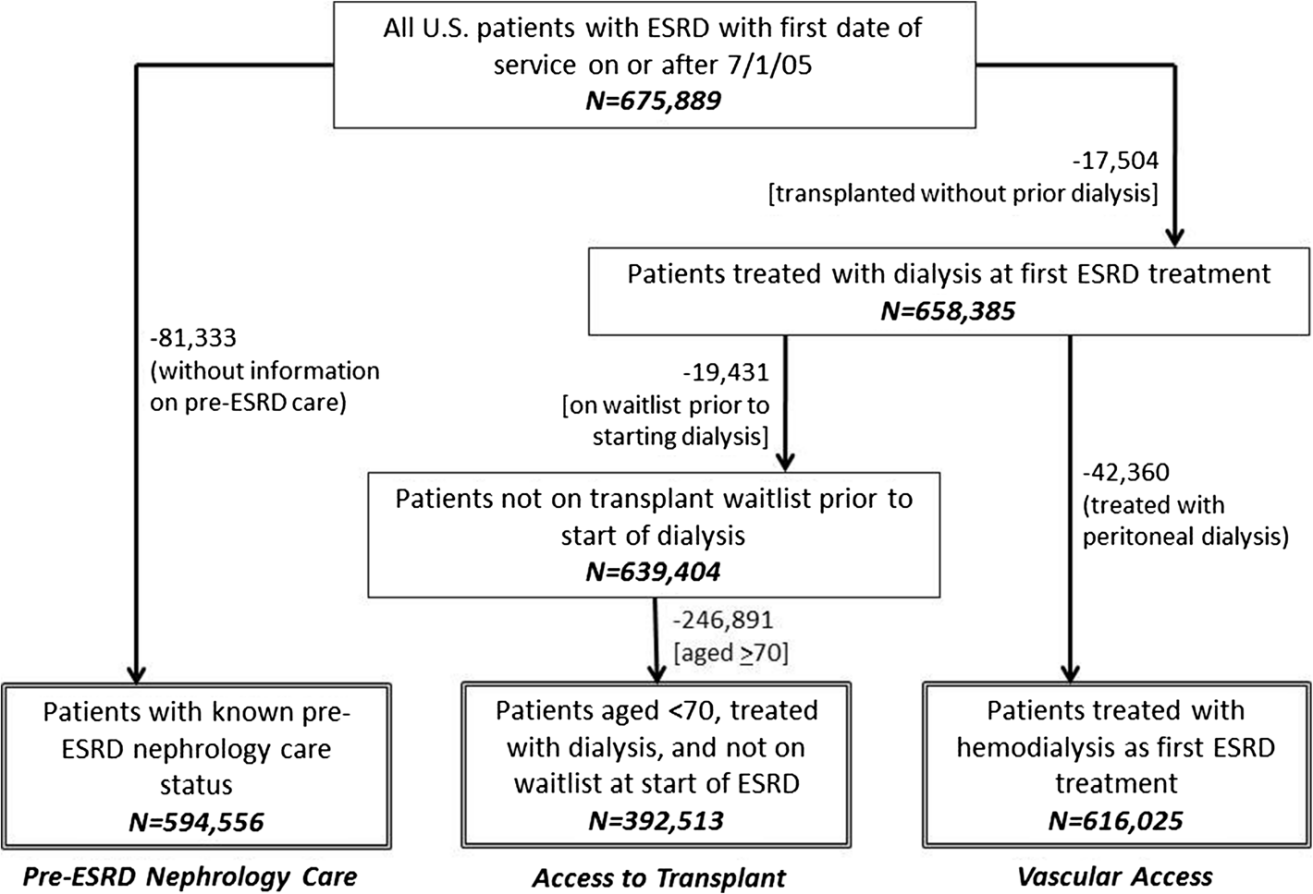
**Selection of analytic populations for examination of the association of attributed cause of ESRD with pre-ESRD nephrology care, access to transplant, and vascular access, among U.S. ESRD patients initiating treatment 7/05–9/11.** Numbers by arrows represent the numbers of patients excluded by indicated criteria; numbers in boxes represent those remaining after prior exclusions.

Data from the most recent (2005) version of the CMS-2728, completed on all treated U.S. incident ESRD patients, were obtained from the United States Renal Data System (USRDS) [15]. Patient consent was not required or possible in this secondary analysis of de-identified data, and the Emory Institutional Review Board approved the study protocol (IRB00063645). A total of 675,889 incident ESRD patients initiated treatment from 7/1/05 to 9/30/11 with available data on primary attributed cause of ESRD. Of these, 81,333 (12.0%) had unknown pre-ESRD nephrology care status and were excluded from these analyses (Figure 1). For analyses of measures of access to kidney transplantation (informed of transplant options and placement on the deceased donor kidney waitlist), those who received transplants without prior dialysis (n = 17,504), were placed on the waitlist prior to starting dialysis (*n* = 19,431), or were aged ≥70 years (*n* = 246,891) were excluded from the 675,889 ESRD patients, leaving 392,513 for analyses (Figure 1). For analyses of permanent vascular access, those who received transplants without prior dialysis (*n* = 17,504) or treated with peritoneal dialysis instead of hemodialysis (*n* = 42,360) were excluded, leaving 616,025 for analysis (Figure 1).

Primary attributed cause of ESRD, quality-of-care measures (nephrology care prior to ESRD, being informed of transplant options, and vascular access at first dialysis), race/ethnicity, insurance, and clinical factors were all obtained from the CMS-2728 through the USRDS. Information on placement on the deceased donor kidney transplant waitlist was obtained from United Network for Organ Sharing (UNOS) data through the USRDS.

### Study variables

#### Attributed cause of ESRD

The exposure of interest was the primary attributed cause of ESRD, which was defined by International Classification of Diseases (ICD)-9 codes listed on the CMS-2728. LN-ESRD was defined as ESRD attributed to secondary glomerulonephritis due to SLE (CMS-2728 ICD-9 code = 710.0). ESRD due to other GN was included as a separate category for comparison with LN-ESRD due to potential similarities in patient population, disease course, and treatment. GN-ESRD was defined by CMS-2728 ICD-9 codes for glomerulonephritis (582.9, 582.1, 583.1, 583.21, 583.22, 583.81, 583.82, 583.4, 580.0, and 582.0) or secondary glomerulonephritis/vasculitis (excluding LN-ESRD; 287.0, 710.1, 283.11, 446.0, 446.4, 583.92, 446.20, 446.21, and 583.91). All other causes of ESRD, which served as the referent group in main analyses, included all other ICD-9 codes as listed on the CMS-2728. Since the majority of incident ESRD in the United States is attributed to diabetes or hypertension (72%) or GN (6%) [15] and the remaining attributed causes represent a fairly diverse group of ESRD etiologies such as cystic kidney disease, we conducted sensitivity analyses including only patients with ESRD attributed to diabetes (250.4x) or hypertension or large vessel disease (CMS-2728 ICD-9 code = 403.91, 440.1, 583.81, 593.83)—representing typical U.S. ESRD patients—in the referent group.

#### Quality-of-care measures

The outcomes of interest were quality-of-care measures related to pre-ESRD care, access to transplant, and permanent vascular access placement. Pre-ESRD nephrology care was defined by an answer of “Yes” to item 18b on the CMS-2728: “Prior to ESRD therapy: was the patient under the care of a nephrologist?” Duration of pre-ESRD care (>12 months, 6–12 months, <6 months, or none) was also recorded. Whether patients were informed of transplant option was defined by CMS-2728 item 26: “Has patient been informed of kidney transplant options?” with possible responses of “Yes” and “No.” Date of placement on the deceased donor transplant waitlist was determined from UNOS data and used to calculate time to placement on the transplant waitlist (date of placement – first ESRD service date). Censoring occurred at death or at the end of follow-up (9/30/11; median follow-up, 1.9 years). Finally, vascular access was determined from CMS-2728 item 18d: “What access was used on first outpatient dialysis?” with possible responses of “AVF,” “Graft,” “Catheter,” and “Other” and two additional prompts for maturing permanent accesses in place (“Is maturing AVF present?” and “Is maturing graft present?”). Permanent vascular access was defined as AVF or graft used or in place on first dialysis.

#### Other variables

Incident age and sex were obtained from the USRDS patient demographics file. Race/ethnicity (defined as white, black, Hispanic, and other), insurance prior to ESRD (defined as private, Medicaid, none, or other), smoking status, BMI, presence of comorbid conditions, and serum albumin and hemoglobin at the start of ESRD were obtained from the CMS-2728. Recovery of renal function, from the patient history file, was defined as any discontinuation of renal replacement therapy over the course of ESRD, regardless of whether treatment was later continued.

### Statistical analysis

Patient characteristics including sociodemographics and clinical factors were summarized overall and by attributed cause (LN-ESRD, GN-ESRD, and other ESRD). Quality-of-care measures were summarized overall and by incident year, with tests for trend. Odds ratios (ORs) and confidence intervals (CIs) for the associations between dichotomous outcomes (pre-ESRD nephrology care, informed of transplant options, and permanent vascular access placement) were estimated with multivariable logistic regression models. For placement on the transplant waitlist, time-to-event analyses were used. To address potential non-proportionality [by tests of Schoenfeld residuals (*P* < 0.001) and examination of log-log curves], hazard ratios (HRs) and CIs were obtained from multivariable Cox proportional hazards models before and after 1 year of ESRD treatment. Factors that were associated with both attributed cause and quality-of-care measures and were not thought *a priori* to be mediators of the association were considered potential confounders. Sensitivity analyses (*i*) with diabetes and hypertension as the referent group (see above); (*ii*) removing those who recovered renal function [31]; (*iii*) with further adjustment for albumin (missing on 23% of patients); and (*iv*) with allowances for non-linear associations with age and interactions between age and sex; as well as measure-specific sensitivity analyses, were also conducted. Stata v. 13 (StataCorp, College Station, TX) was used for all analyses.

## Results

### Characteristics of the study population by attributed ESRD cause

Patients with ESRD due to LN had a mean age of 40 years and were, on average, 14 and 24 years younger than patients with ESRD due to other glomerulonephritis (GN) and patients with ESRD due to other causes, respectively (Table 1). The majority of LN-ESRD patients were female, compared to fewer than half of GN-ESRD and other ESRD patients (Table 1). Similarly, half of LN-ESRD patients were black, compared to approximately one-quarter of GN-ESRD and other ESRD patients (Table 1). Cardiovascular disease was far more common in other ESRD patients than among LN- or GN-ESRD patients, and those with LN-ESRD were less likely to report smoking and also had lower body mass index (BMI) and lower levels of albumin and hemoglobin than those with GN-ESRD or other ESRD (Table 1).

**Table 1:** **Characteristics of U.S. ESRD patients with attributed causes of lupus nephritis, other types of glomerulonephritis, and all other causes, 7/05–9/11**

**Characteristic**	**Overall**	Attributed cause of ESRD*		
		**LN**	**Other GN**	**All other**
*N*	*675,889*	*6,594*	*51,537*	*617,758*
**Sociodemographic**				
Age, years, mean (SD)	62.5 (16.0)	39.6 (15.4)	53.9 (18.9)	63.4 (15.3)
Sex, %				
Female	43.7%	81.1%	40.7%	43.5%
Male	56.3%	18.9%	59.3%	56.5%
Race/ethnicity, %				
White	52.9%	24.7%	57.8%	52.7%
Black	28.1%	49.7%	22.5%	28.3%
Hispanic	13.4%	17.7%	12.3%	13.4%
Other	5.7%	7.9%	7.4%	5.6%
Insurance at ESRD start, %				
Private	31.3%	37.4%	41.8%	30.3%
Medicaid	24.5%	32.8%	18.6%	24.9%
Medicare/other	36.8%	18.4%	30.0%	37.5%
None	7.5%	11.5%	9.6%	7.3%
**Clinical**				
Smoking, %				
No	93.8%	95.7%	92.7%	93.9%
Yes	6.2%	4.3%	7.3%	6.1%
BMI, kg/m^2^, mean (SD)	28.9 (7.8)	26.9 (7.4)	28.1 (7.5)	29.0 (7.8)
Hypertension, %				
No	15.4%	16.4%	17.0%	15.3%
Yes	84.6%	83.6%	83.0%	84.7%
CVD, %				
No	57.8%	81.4%	75.8%	56.0%
Yes	42.2%	18.6%	24.2%	44.0%
Albumin, g/dl, mean (SD)	3.1 (0.7)	2.9 (0.8)	3.2 (0.8)	3.1 (0.7)
Hemoglobin, g/dl, mean (SD)	10.0 (1.7)	9.5 (1.7)	10.0 (1.8)	10.0 (1.6)
Recovery of renal function, %				
No	95.5%	93.1%	95.9%	95.5%
Yes	4.5%	6.9%	4.1%	4.5%

LN, lupus nephritis; GN, glomerulonephritis; BMI, body mass index; CVD, cardiovascular disease, including pericarditis; p-y, person-years.

*P < 0.001 for all comparisons across attributed cause, by ANOVA, chi-square, or log-rank test.

### Association of attributed cause of ESRD with quality-of-care measures

#### Pre-ESRD care

Overall, about two-thirds of U.S. ESRD patients received pre-ESRD nephrology care, with LN-ESRD (71%) and GN-ESRD (69%) patients more likely to receive pre-ESRD care than other ESRD (65%) patients (Table 2). LN- and GN-ESRD patients were also more likely to receive greater duration of pre-ESRD care than other ESRD patients (>12 months, 36% and 35% vs. 27%; >6 months, 57% and 56% vs. 51%; *P* < 0.001 for both; not shown in table). Receipt of pre-ESRD care among incident patients increased slightly from 2005 to 2011 for each attributed cause, although the trend was not statistically significant for LN-ESRD (Table 2). After adjustment for potential sociodemographic and clinical confounders, those with LN-ESRD were nearly 70% more likely than other ESRD patients to receive pre-ESRD care, whereas GN-ESRD patients were only about 20% more likely to receive this care (Table 3). The associations were slightly stronger for longer duration [pre-ESRD care ≥12 vs. <12 months: LN-ESRD, OR = 1.82, (95% CI, 1.70–1.92); GN-ESRD, OR = 1.42 (95% CI, 1.39–1.45)] and weaker for shorter duration [pre-ESRD care ≥6 vs. <6 months: LN-ESRD, OR = 1.50 (95% CI, 1.42–1.59); GN-ESRD, OR = 1.21 (95% CI, 1.18–1.23)]. Results from other sensitivity analyses were similar to the primary analyses (Table 4).

**Table 2:** **Attainment of quality-of-care measures by cause of ESRD (lupus nephritis, other glomerulonephritis, and all other causes) and by incident year, among U.S. ESRD patients initiating treatment 7/05–9/11**

Quality-of-care measure*	**Entire follow-up (7/05–9/11)**	**Incident year**							***P***_***trend***_
		**2005**	**2006**	**2007**	**2008**	**2009**	**2010**	**2011**	
**Pre-ESRD nephrology care, %**									
All ESRD (*n* = 594,556)	65.7%	65.9%	65.6%	65.0%	65.0%	65.5%	65.9%	67.7%	*<0.001*
ESRD attributed to:									
Lupus nephritis (*n* = 5,939)	71.1%	72.2%	70.2%	71.7%	70.3%	73.1%	68.9%	72.3%	*0.96*
Other glomerulonephritis (*n* = 48,031)	69.3%	69.3%	69.1%	67.7%	69.2%	69.0%	70.4%	71.2%	*0.002*
All other causes (*n* = 540,586)	65.3%	65.5%	65.2%	64.6%	64.6%	65.1%	65.5%	67.4%	*<0.001*
**Informed of transplant options, %**									
All ESRD (*n* = 392,513)	78.9%	76.9%	76.2%	76.6%	78.0%	80.5%	81.4%	82.4%	*<0.001*
ESRD attributed to:									
Lupus nephritis (*n* = 5,619)	84.8%	87.0%	82.6%	82.6%	84.8%	85.9%	86.5%	85.8%	*0.07*
Other glomerulonephritis (*n* = 32,325)	83.6%	82.3%	82.1%	81.6%	83.6%	84.5%	85.1%	87.0%	*<0.001*
All other causes (*n* = 354,569)	78.3%	76.1%	75.5%	76.0%	77.4%	80.1%	81.1%	82.0%	*<0.001*
**Placement on the kidney transplant waitlist, events/1000 p-y**									
All ESRD (*n* = 392,513)	97	83	88	91	99	114	122	95	*<0.001*
ESRD attributed to:									
Lupus nephritis (*n* = 5,619)	206	194	177	208	210	230	263	180	*0.07*
Other glomerulonephritis (*n* = 32,325)	203	162	181	190	211	240	277	232	*<0.001*
All other causes (*n* = 354,569)	86	74	78	80	88	102	108	82	*<0.001*
**Permanent vascular access used or in place at ESRD start, %**									
All ESRD (*n* = 616,025)	35.9%	37.4%	36.7%	35.4%	34.3%	35.1%	36.4%	37.4%	*0.89*
ESRD attributed to:									
Lupus nephritis (*n* = 5,624)	24.4%	22.3%	25.7%	23.9%	23.5%	24.3%	25.5%	25.3%	*0.45*
Other glomerulonephritis (*n* = 41,824)	37.7%	40.3%	38.6%	37.4%	36.1%	36.7%	37.1%	39.6%	*0.83*
All other causes (*n* = 568,577)	35.9%	37.3%	36.7%	35.4%	34.2%	35.0%	36.5%	37.4%	*0.11*

ESRD, end-stage renal disease; p-y, patient year. *P < 0.001 for all overall and within-year comparisons of measures across attributed cause of ESRD.

**Table 3:** **Risk ratios for attributed causes of lupus nephritis and other glomerulonephritis vs. other causes of ESRD, among U.S. ESRD patients initiating treatment 7/05–9/11**

**Quality of care measure**	**Risk ratio for attributed cause of ESRD (95% CI)**		
	**Unadjusted**	Adjusted*	
		**Sociodemographic**	**Sociodemographic and clinical**
**Pre-ESRD care, yes vs. no (odds ratio)**			
All other causes	1.00 (ref)	1.00 (ref)	1.00 (ref)
Lupus nephritis	1.64 (1.54–1.74)	1.51 (1.42–1.61)	1.68 (1.57–1.78)
Other glomerulonephritis	1.26 (1.23–1.28)	1.19 (1.17–1.22)	1.22 (1.19–1.24)
**Informed of transplant options, yes vs. no (odds ratio)**			
All other causes	1.00 (ref)	1.00 (ref)	1.00 (ref)
Lupus nephritis	1.10 (1.02–1.19)	1.09 (1.01–1.18)	1.10 (1.02–1.19)
Other glomerulonephritis	1.19 (1.15–1.23)	1.21 (1.17–1.25)	1.19 (1.15–1.23)
**Time to placement on the kidney transplant waitlist (hazard ratio)**			
***In 1st year of ESRD***			
All other causes	1.00 (ref)	1.00 (ref)	1.00 (ref)
Lupus nephritis	2.29 (2.15–2.43)	1.47 (1.39–1.57)	1.42 (1.34–1.52)
Other glomerulonephritis	2.73 (2.66–2.80)	2.00 (1.95–2.05)	1.91 (1.86–1.96)
***After 1st year of ESRD***			
All other causes	1.00 (ref)	1.00 (ref)	1.00 (ref)
Lupus nephritis	2.45 (2.29–2.63)	1.60 (1.49–1.72)	1.56 (1.45–1.67)
Other glomerulonephritis	1.90 (1.84–1.97)	1.45 (1.40–1.50)	1.39 (1.35–1.44)
**Permanent vascular access used/in place, yes vs. no (odds ratio)**			
All other causes	1.00 (ref)	1.00 (ref)	1.00 (ref)
Lupus nephritis	0.57 (0.53–0.61)	0.58 (0.55–0.62)	0.63 (0.59–0.67)
Other glomerulonephritis	1.07 (1.05–1.10)	1.07 (1.05–1.10)	1.10 (1.07–1.12)

*Sociodemographic: age, race, sex, and insurance; clinical: body mass index, cardiovascular disease (including pericarditis), and hemoglobin.

**Table 4:** **Risk ratios for attributed causes of lupus nephritis and other glomerulonephritis vs. other causes of ESRD, among U.S. ESRD patients initiating treatment 7/05–9/11: sensitivity analyses**

**Attributed cause of ESRD**	Risk ratio* for attributed cause of ESRD (95% CI)				
	**Diabetes and hypertension only as referent group**	**Additional adjustment for albumin**	**Among patients who never recovered renal function**	**Additional adjustment for age-squared**	**Additional adjustment for age × sex**
**Pre-ESRD care, yes vs. no (odds ratio)**					
All other causes	1.00 (ref)	1.00 (ref)	1.00 (ref)	1.00 (ref)	1.00 (ref)
Lupus nephritis	1.56 (1.46–1.66)	2.02 (1.88–2.17)	1.69 (1.59–1.81)	1.71 (1.61–1.82)	1.55 (1.46–1.65)
Other glomerulonephritis	1.06 (1.04–1.09)	1.20 (1.17–1.23)	1.20 (1.18–1.23)	1.23 (1.20–1.25)	1.22 (1.19–1.24)
**Informed of transplant options, yes vs. no (odds ratio)**					
All other causes	1.00 (ref)	1.00 (ref)	1.00 (ref)	1.00 (ref)	1.00 (ref)
Lupus nephritis	0.98 (0.91–1.06)	1.16 (1.06–1.26)	1.11 (1.02–1.20)	1.13 (1.04–1.22)	1.09 (1.01–1.18)
Other glomerulonephritis	1.07 (1.03–1.10)	1.19 (1.14–1.23)	1.20 (1.16–1.24)	1.20 (1.16–1.24)	1.19 (1.15–1.23)
**Time to placement on the kidney transplant waitlist (hazard ratio)**					
***In 1st year of ESRD***					
All other causes	1.00 (ref)	1.00 (ref)	1.00 (ref)	1.00 (ref)	1.00 (ref)
Lupus nephritis	1.53 (1.43–1.63)	1.53 (1.43–1.64)	1.50 (1.41–1.59)	1.44 (1.35–1.53)	1.39 (1.31–1.48)
Other glomerulonephritis	2.01 (1.95–2.06)	1.86 (1.81–1.92)	1.88 (1.83–1.93)	1.93 (1.88–1.98)	1.91 (1.86–1.96)
***After 1st year of ESRD***					
All other causes	1.00 (ref)	1.00 (ref)	1.00 (ref)	1.00 (ref)	1.00 (ref)
Lupus nephritis	1.56 (1.45–1.67)	1.69 (1.57–1.83)	1.66 (1.54–1.78)	1.57 (1.46–1.69)	1.52 (1.42–1.63)
Other glomerulonephritis	1.36 (1.31–1.41)	1.37 (1.32–1.42)	1.38 (1.33–1.43)	1.41 (1.36–1.46)	1.39 (1.34–1.44)
**Permanent vascular access used/in place, yes vs. no (odds ratio)**					
All other causes	1.00 (ref)	1.00 (ref)	1.00 (ref)	1.00 (ref)	1.00 (ref)
Lupus nephritis	0.57 (0.53–0.60)	0.63 (0.59–0.67)	0.64 (0.60–0.69)	0.73 (0.68–0.77)	0.61 (0.57–0.65)
Other glomerulonephritis	0.97 (0.95–0.99)	1.10 (1.07–1.12)	1.09 (1.06–1.11)	1.14 (1.12–1.17)	1.10 (1.07–1.12)

*Adjusted for age, race, sex, insurance, body mass index, cardiovascular disease (including pericarditis), and hemoglobin.

#### Access to transplant

Overall, 79% of U.S. ESRD patients were informed of transplant options at the start of ESRD, with 85%, 84%, and 78% of patients with ESRD due to LN, GN, and other causes being informed. Absolute increases of about 5% in being informed of transplant options were seen over study follow-up for all incident ESRD patients, although the trend was marginally statistically significant for LN-ESRD patients (*P* = 0.07; Table 2). With adjustment, ESRD patients with LN and GN were 10% and 19% more likely than those with other causes to be informed of the transplant options (Table 3). Estimates were nearly identical when patients aged ≥70 years were included, to account for increasing transplantation in older adults [32] [LN-ESRD, OR = 1.10 (95% CI, 1.02–1.19); GN-ESRD, OR = 1.19 (95% CI, 1.15–1.23)], and results were similar in other sensitivity analyses as well (Table 4).

**Figure 2:**
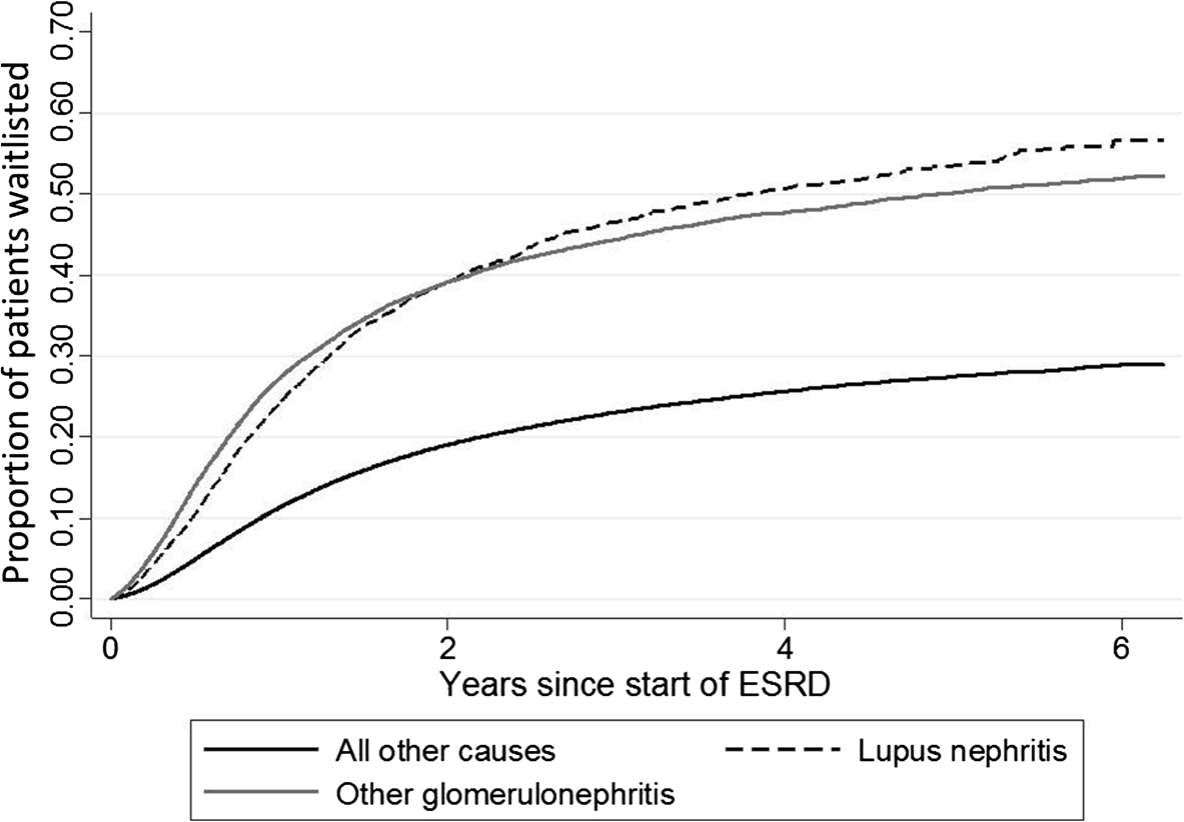
**Cumulative incidence of placement on the deceased donor kidney transplant waitlist among U.S. ESRD patients initiating treatment 7/05–9/11, by attributed cause of ESRD.** *P* < 0.001 by log-rank.

Incidence of placement on the kidney transplant waitlist was 97 per 1000 patient-years overall but was more than twice as high among LN-ESRD and GN-ESRD patients as compared to other ESRD patients (Table 2). Placement on the waitlist increased over time among patients with all causes of ESRD, although the trend was marginally statistically significant for LN-ESRD patients (Table 2). Time to placement was similar among ESRD patients with LN and GN but was much shorter among both groups of patients compared to other ESRD patients (Figure 2). Adjusted analyses showed that the rate of placement on the waitlist among LN-ESRD patients was 42% higher than that among other ESRD patients in the first year of ESRD; this relatively increased rate was even higher (56%) after the first year (Table 3). In comparison, GN-ESRD patients had nearly twice the rate of placement on the kidney transplant waitlist as other ESRD patients in the first year but only a ~40% higher rate after the first year (Table 3). When those aged ≥70 years were included, results were nearly identical [within first year: LN-ESRD, OR = 1.42 (95% CI, 1.34–1.52); GN-ESRD, OR = 1.91 (95% CI, 1.86–1.96); after first year: [LN-ESRD, OR = 1.56 (95% CI, 1.45–1.67); GN-ESRD, OR = 1.39 (95% CI, 1.35–1.44)]. Results in other sensitivity analyses were also similar (Table 4). When being transplanted without placement on the waitlist (which occurred in 1.6%, 1.2%, and 1.0% of patients with ESRD due to LN, GN, and all other causes, respectively) was combined with placement on the waitlist as an outcome, associations were only slightly attenuated (data not shown).

#### Permanent vascular access

**Figure 3:**
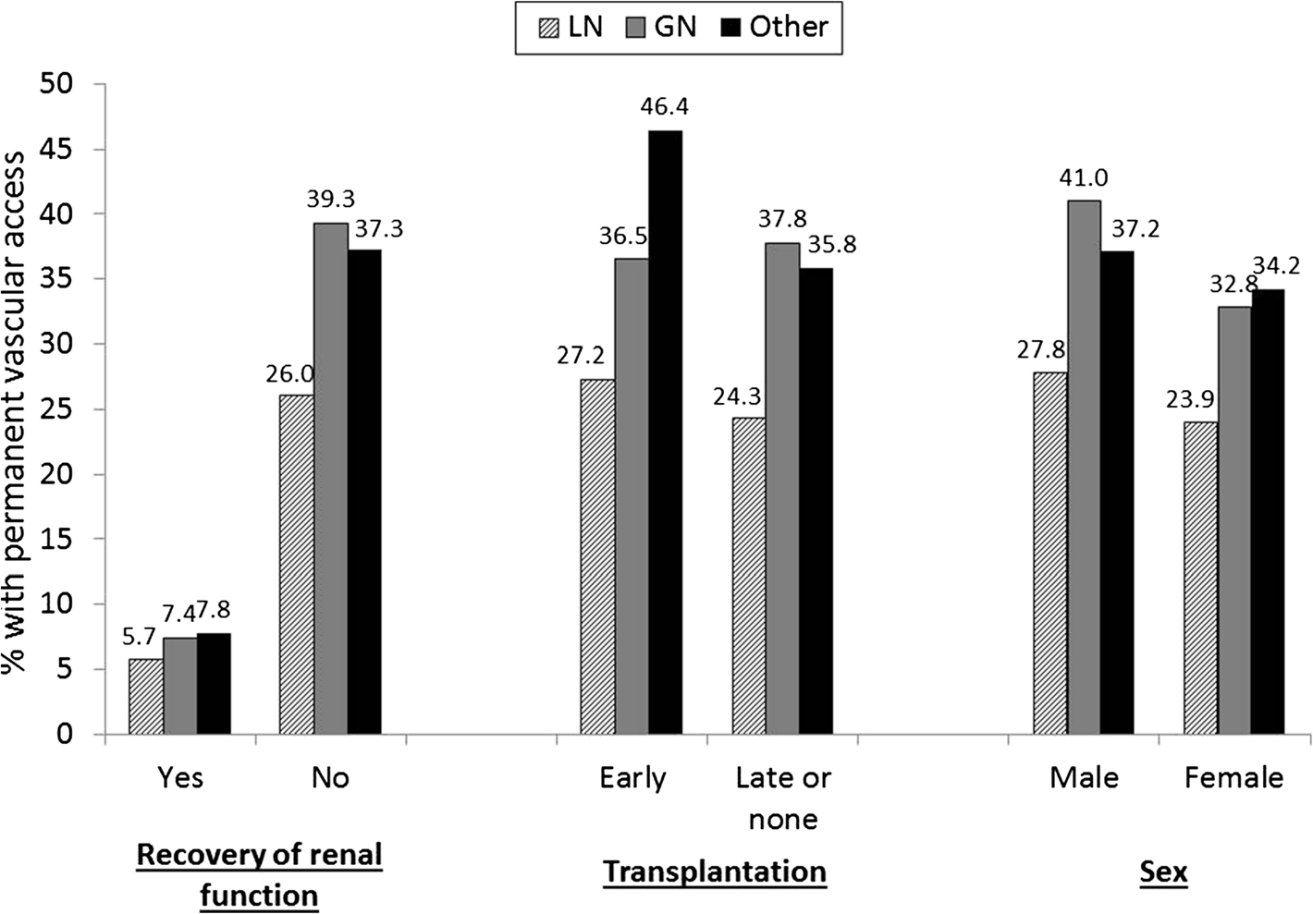
**Permanent vascular access placement by patient characteristics, among U.S. ESRD patients initiating treatment 7/05–9/11, by attributed cause of ESRD.** Recovery of renal function is defined as recovery occurring at any time during treatment, regardless of whether patient returned to dialysis; early transplant is defined as a transplant within 1 year of ESRD start.

More than one-third of all dialysis patients had a permanent vascular access in place at the start of treatment, but fewer than one-quarter of LN-ESRD patients had a fistula or graft in place (Table 2). There were no differences over time in permanent vascular access placement overall or by cause of ESRD (Table 2). With adjustment, LN-ESRD patients remained nearly 40% less likely than other ESRD patients to have a permanent vascular access used or in place at first dialysis, whereas GN-ESRD patients were 10% more likely than other ESRD patients to have a permanent vascular access (Table 3). Results were similar in sensitivity analyses (Table 4). Placement of permanent access was far less common among patients who recovered function at any point, compared to those who did not recover function, regardless of attributed cause (Figure 3). Patients with other causes of ESRD who had early transplants (within 1 year of ESRD start) were more likely than similar patients who had not received a transplant within 1 year of ESRD start to have a permanent vascular access (*P* < 0.001), but this was not true among LN-ESRD or GN-ESRD patients (Figure 3). Among those with early transplants and with full adjustment, both LN-ESRD (OR = 0.66, 95% CI, 0.48–0.92) and GN-ESRD (OR = 0.83, 95% CI, 0.74–0.93) patients were less likely than other ESRD patients to have a permanent vascular access in place at start of ESRD. For all attributed cause groups, males were more likely than females to have a permanent vascular access used or in place at the start of ESRD (*P* < 0.05 for all causes; Figure 3).

## Discussion

Compared to other ESRD patients, LN-ESRD patients represent a group that may receive greater clinical attention due their underlying SLE and young age. Our results showed that, indeed, receipt of pre-ESRD nephrology care and access to the kidney transplant waitlist were higher among LN-ESRD vs. other ESRD patients. After adjustment for differences across the patient populations—including age, sex, race, and insurance, as well as clinical characteristics—LN-ESRD patients remained more likely than other ESRD patients to have had pre-ESRD care, to be informed of transplant options at the start of ESRD, and to be placed on the deceased donor kidney transplant waitlist while on dialysis. These patterns were similar to those seen in the comparison of GN-ESRD to other ESRD patients. However, only about one-quarter of LN-ESRD patients had a permanent vascular access in place at the start of dialysis, and LN-ESRD patients remained strikingly less likely than either GN-ESRD or other ESRD patients to have a permanent vascular access in place at the start of dialysis, accounting for patient characteristics.

While LN-ESRD patients were nearly 70% more likely than other ESRD patients to have pre-ESRD nephrology care after adjustment for differences in the populations, nearly one-third of patients with SLE and LN progressed to ESRD without ever having seen a nephrologist. Further, the percentage of patients receiving pre-ESRD care generally increased slightly over time among most ESRD patients, but not among those with LN-ESRD. Progression of LN is much faster among black patients [33,34], who are overrepresented in LN and SLE and may also be less likely to access care early, which may make nephrology referral prior to development of ESRD more difficult. It is also possible that progression to ESRD may be quite rapid among some kidney disease patients [35]. Particularly among SLE patients, due to the relapsing-remitting nature of SLE and LN, potentially involving sudden renal flares [36], it may be difficult in some cases to refer to nephrology prior to the urgent need for dialysis. However, it is likely that such presentations are rare and that, with greater attention to signs of renal damage and dysfunction (biopsy-proven GN, hematuria, proteinuria, and reduced glomerular filtration rate) among patients with adequate access to SLE care, lack of pre-ESRD nephrology care among LN-ESRD patients could be greatly reduced.

Being informed of transplant options and especially placement on the kidney transplant waitlist increased over study follow-up for patients with all attributed causes of ESRD; these secular trends have been previously noted in the U.S. LN-ESRD population [27,28]. Changes in criteria for placement on the kidney transplant waitlist that have reduced racial disparities [37,38] may have resulted in greater access to the waitlist among the minority LN-ESRD population. Additionally, increasing evidence that transplant outcomes among LN-ESRD and other ESRD patients appear to be equivalent [39-41] may have contributed to this increase. While both LN-ESRD and GN-ESRD patients were more likely to be placed on the kidney transplant waitlist than patients with other ESRD, the association was even stronger after the first year of ESRD among LN-ESRD patients, which may reflect recommendations to wait to transplant LN-ESRD patients to allow SLE activity to decrease [42,43]. However, whether such delays are necessary or potentially even detrimental in this population remains in question [44].

Despite a national quality initiative program initially implemented in 2003–2004 to increase the use of AVFs [45], most U.S. ESRD patients started dialysis without a permanent vascular access in place, and the percentage with a permanent vascular access at the start of dialysis did not increase appreciably over study follow-up for any ESRD group we examined—mirroring recent reports that show that, while placements of fistulae have increased, the use of temporary catheters has not decreased substantially [46]. LN-ESRD patients were far less likely than either GN-ESRD or other ESRD patients to have a permanent access, a gap that has been noted previously in pediatric SLE patients [47,48]. While female sex may be associated with more difficulty placing AVFs [49], greater likelihood of body image issues associated with permanent vascular access [50,51], and generally increased complexity (*e.g*., contraception and fertility concerns approaching dialysis), accounting for the female predominance in the LN-ESRD population did not change the results. Further, we found that males with LN-ESRD were only slightly more likely than females with LN-ESRD to have a permanent vascular access. The possibility that providers may skip permanent vascular access among patients who may receive an early transplant was also not supported by our findings. Having a permanent vascular access in place at the start of dialysis was as or more likely among those patients who received a transplant within a year of ESRD start than other ESRD patients. Greater anticipated recovery from ESRD among SLE patients could play a role [31], but we found LN-ESRD patients were less likely than other patients to have a permanent access in place at dialysis start, regardless of evidence of recovery of renal function. History of multiple, prolonged intravenous treatments in SLE patients could play a role in decisions not to refer for vascular access surgery, as could hypercoagulable states in SLE patients, particularly in the setting of anti-phospholipid syndrome [43]; however, neither of these possibilities could be examined with available data. Finally, many barriers to vascular access that have been noted in the overall ESRD population, including fear of needles, issues of coping with thoughts of imminent dialysis, and the threat of potential physical deformity due to vascular access [51], may be particularly salient in the younger LN-ESRD population. Further, follow-up may be less consistent in this population, preventing providers from discussing the importance of creating a permanent vascular access.

Similar to the population with ESRD secondary to sickle cell disease [29], another young, primarily minority patient population with multiple providers, the LN-ESRD population showed substantial gaps in placement of permanent vascular access for hemodialysis. However, unlike the sickle cell population, LN-ESRD patients were more likely than the general ESRD population to receive pre-ESRD care. Such disparate patterns of adequacy of care in LN-ESRD could be the result of so-called “silos” of care, in which there is lack of communication and coordination among specialty providers and a loss of patient-centeredness [52]. Lack of direct communication between nephrologists and rheumatologists and the general lack of guidelines in rheumatology to address preparation for ESRD [30] may discourage the rheumatologist from actively participating in certain treatment decisions for their LN-ESRD patients. While rheumatologists may refer appropriately to nephrologists, they may leave discussions of specific preparation for transplantation and dialysis to nephrologists; in turn, nephrologists may assume that rheumatologists are coordinating the overall care of the patient approaching LN-ESRD and spend less time discussing ESRD preparation with these patients. Such gaps in communication in this critical period could lead to less preparedness for the initiation of ESRD (*e.g*., placement of permanent vascular access) but greater access to treatment options (*e.g*., placement on the kidney transplant waitlist) after the start of ESRD, when SLE activity may “burn out” [42].

This study has several limitations. The USRDS does not capture non-Medicare-eligible individuals who have untreated ESRD, including some undocumented residents. Also, attribution of ESRD cause on the CMS-2728 has unknown validity; only one small validation study has been published [53], suggesting low sensitivity. There is the potential for selection bias due to missing data (12.0%) in analyses of pre-ESRD care. Misclassification of quality of care measures on the CMS-2728 is also possible, particularly provision of information about kidney transplant [54], due to variability in provider knowledge about patients. Many potential confounders, such as hemoglobin and albumin, may be the result of the adequacy of care, rather than a factor that leads to adequate care. Such factors may serve as mediating factors rather than confounders, leading to potential overadjustment, although we found that crude and adjusted results generally did not differ substantially. As with any observational study, residual confounding is possible. For example, although we excluded patients aged ≥ 70 and adjusted for CVD in the primary analysis, we do not have specific, more granular data on kidney transplant eligibility, which is likely to be higher among the LN-ESRD patients and could be a confounder of the associations with kidney transplant access measures. We also could not adjust for acute kidney injury status leading to ESRD, which might be a marker of fast progression and provider inability to intervene prior to start of ESRD, although we were able to examine associations among those who never recovered renal function. This study also has several powerful strengths, including the capture of all U.S. patients treated for ESRD, limited loss to follow-up due to universal coverage of ESRD services by CMS, and the completion of the CMS-2728 for all treated patients.

## Conclusions

There is room for improvement in all quality-of-care measures among SLE patients approaching ESRD. While patients with LN-ESRD are more likely to receive pre-ESRD care and have better access to transplant than patients with ESRD due to other causes, they are far less likely than their counterparts to have a permanent vascular access in place for dialysis. Further studies are warranted to specifically examine patient-, provider-, and system-level barriers to permanent vascular access placement and to estimate the morbidity and mortality associated with temporary access in the LN-ESRD population, as well as to examine potential barriers to adequate ESRD care in patients with SLE.
